# Supplementary material for: MetaRibo-Seq measures translation in microbiomes
Source: Nat Commun. 2020 Jun 29;11:3268. doi: 10.1038/s41467-020-17081-z (PMC7324362; doi:10.1038/s41467-020-17081-z)
Supplement: Supplementary file 10 — Supplementary Data 7 [file 41467_2020_17081_MOESM10_ESM.zip › File2/Confidence_High_Taxonomy/31924_out.krona.html]

Javascript must be enabled to view this page.

members
magnitude
magnitudeUnassigned
count
unassigned
taxon
rank

31924\_out

10

SRS017521\_contig\_number\_contig-100\_4655.50913SRS021153\_contig\_number\_contig-100\_3765.3765SRS054956\_contig\_number\_contig-100\_8139.8139SRS075947\_contig\_number\_contig-100\_6417.6418SRS075957\_contig\_number\_contig-100\_2180.2180SRS077312\_contig\_number\_contig-100\_13082.13082SRS077502\_contig\_number\_contig-100\_10207.10207SRS140492\_contig\_number\_7617SRS147151\_contig\_number\_contig-100\_2749.2750SRS147377\_contig\_number\_contig-100\_9645.9646
150


SRS012902\_contig\_number\_contig-100\_3629.3629SRS098717\_contig\_number\_contig-100\_310.310SRS1041031\_contig\_number\_contig-100\_676.677SRS104285\_contig\_number\_contig-100\_2299.2300SRS105082\_contig\_number\_81SRS143427\_contig\_number\_contig-100\_5237.5237SRS146996\_contig\_number\_2966
7
superkingdom
140
2

6
phylum
1239

186801
class
5

186802
order
5

424536
1
family

genus
1
270497

species
1

SRS011239\_contig\_number\_2
1946289

family
1
186806

1730
genus
1

species
1

SRS143876\_contig\_number\_14738
39485


SRS147919\_contig\_number\_contig-100\_5831.5831
186803
1
family

family
2
541000

2
genus
216851

853

SRS018313\_contig\_number\_5SRS1041134\_contig\_number\_9680
2
species

class
1
91061

1
order
186826

1300
family
1

genus
1
1301

species
1
1305

SRS058808\_contig\_number\_65

203691
1
phylum

203692
class
1

136
1
order

137
1
family

157
1
genus

409322

SRS143310\_contig\_number\_1504
1
species

1224
phylum
125

SRS893342\_contig\_number\_contig-100\_320.321
1

7
subphylum
68525

29547
class
1

213849
order
1

1
family
72294

194
genus
1

species
1

SRS143310\_contig\_number\_contig-100\_1457.40402
199

6
class
28221

213115
6
order

6
family
194924

872
3
genus

species
1
159728

SRS148196\_contig\_number\_contig-100\_716.57247


SRS147557\_contig\_number\_contig-100\_170.171
167968
1
species

1
species
44742

SRS014235\_contig\_number\_contig-100\_93.93

35832
genus
3

SRS017521\_contig\_number\_47
1


SRS018427\_contig\_number\_2228SRS077127\_contig\_number\_71
35833
species
2

class
37
1236

135625
2
order

712
family
2


SRS148091\_contig\_number\_110
1
724
2
genus


SRS021948\_contig\_number\_21331
1715211
species
1


SRS046717\_contig\_number\_10SRS077646\_contig\_number\_contig-100\_2117.2118
2
91347
order
35

1903411
family
1


SRS148096\_contig\_number\_contig-100\_1814.1815
613
genus
1

8

SRS019968\_contig\_number\_contig-100\_34.34SRS022713\_contig\_number\_contig-100\_9221.30856SRS075398\_contig\_number\_921SRS076876\_contig\_number\_625SRS142712\_contig\_number\_12SRS142890\_contig\_number\_280SRS144639\_contig\_number\_contig-100\_1404.1405SRS971428\_contig\_number\_3190
family
32
543

genus
15
561

SRS021153\_contig\_number\_676SRS023829\_contig\_number\_contig-100\_1743.1743SRS077454\_contig\_number\_51
3


SRS013158\_contig\_number\_contig-100\_16.16SRS023715\_contig\_number\_1081SRS023971\_contig\_number\_contig-100\_1519.1519SRS078242\_contig\_number\_256SRS098827\_contig\_number\_95SRS098881\_contig\_number\_94SRS1041134\_contig\_number\_1867SRS1055043\_contig\_number\_47SRS142781\_contig\_number\_1317SRS142896\_contig\_number\_5414SRS143218\_contig\_number\_2363SRS143342\_contig\_number\_170
562
12
species

genus
1
590

1
species
28901

59201

SRS893341\_contig\_number\_1204
subspecies
1


SRS893259\_contig\_number\_1508
1
3
genus
570

species
1

SRS078260\_contig\_number\_contig-100\_945.945
244366

1
species
573

SRS143070\_contig\_number\_197

544

SRS015794\_contig\_number\_1280SRS146888\_contig\_number\_45
genus
2


SRS018822\_contig\_number\_contig-100\_12953.49099
413496
1
genus

547
2
genus

1

SRS018623\_contig\_number\_737
2
species group
354276

species
1

SRS145308\_contig\_number\_405
2511988


SRS019445\_contig\_number\_337
1262986
1
species

28216
79
class

80840
order
79


SRS142599\_contig\_number\_468
469610
1
species

995019
61
family


SRS015782\_contig\_number\_16366SRS018427\_contig\_number\_2153SRS018427\_contig\_number\_contig-100\_18380.140459SRS054905\_contig\_number\_1765SRS054956\_contig\_number\_contig-100\_581.581SRS076804\_contig\_number\_contig-100\_680.681SRS148424\_contig\_number\_9746
7
61
genus
40544


SRS014235\_contig\_number\_15328SRS014855\_contig\_number\_contig-100\_3009.44823SRS015663\_contig\_number\_13357SRS017701\_contig\_number\_700SRS017745\_contig\_number\_1410SRS018888\_contig\_number\_contig-100\_804.805SRS019397\_contig\_number\_contig-100\_474.474SRS019397\_contig\_number\_contig-100\_877.36626SRS019685\_contig\_number\_8248SRS020233\_contig\_number\_31873SRS024075\_contig\_number\_2407SRS043841\_contig\_number\_63SRS044535\_contig\_number\_10848SRS045004\_contig\_number\_4965SRS047044\_contig\_number\_51SRS048981\_contig\_number\_284SRS050801\_contig\_number\_195SRS051031\_contig\_number\_11840SRS054059\_contig\_number\_112SRS054956\_contig\_number\_contig-100\_1774.39416SRS063813\_contig\_number\_158SRS075021\_contig\_number\_182SRS076756\_contig\_number\_9592SRS077641\_contig\_number\_11348SRS078665\_contig\_number\_49SRS098585\_contig\_number\_contig-100\_1310.1310SRS100907\_contig\_number\_contig-100\_82.83SRS101376\_contig\_number\_11080SRS104036\_contig\_number\_55SRS1041112\_contig\_number\_800SRS104327\_contig\_number\_contig-100\_1547.1548SRS104912\_contig\_number\_33SRS105082\_contig\_number\_8824SRS1055076\_contig\_number\_1193SRS142923\_contig\_number\_4SRS144116\_contig\_number\_contig-100\_331.331SRS144506\_contig\_number\_18426SRS145497\_contig\_number\_2077SRS147271\_contig\_number\_23616SRS147919\_contig\_number\_701SRS148253\_contig\_number\_59SRS893382\_contig\_number\_101
40545
42
species

species
12

SRS015431\_contig\_number\_36391SRS016132\_contig\_number\_17SRS019381\_contig\_number\_2366SRS021948\_contig\_number\_523SRS097889\_contig\_number\_13783SRS105153\_contig\_number\_14343SRS1054716\_contig\_number\_4518SRS143780\_contig\_number\_3335SRS144135\_contig\_number\_267SRS144537\_contig\_number\_contig-100\_20522.162064SRS147425\_contig\_number\_277SRS893369\_contig\_number\_1784
2292075

family
17
80864

1

SRS098824\_contig\_number\_1627
219181
genus
17

12
species

SRS013533\_contig\_number\_12563SRS015650\_contig\_number\_1SRS057886\_contig\_number\_contig-100\_3367.3368SRS063246\_contig\_number\_contig-100\_6759.6760SRS064260\_contig\_number\_7SRS098567\_contig\_number\_contig-100\_129.129SRS142883\_contig\_number\_1449SRS148157\_contig\_number\_6822SRS148193\_contig\_number\_12319SRS148730\_contig\_number\_19SRS149286\_contig\_number\_11SRS893355\_contig\_number\_142
2045302

1658672

SRS018826\_contig\_number\_133SRS045588\_contig\_number\_contig-100\_1.2SRS077245\_contig\_number\_42SRS147871\_contig\_number\_419
species
4

1
phylum
201174

1
class
1760

order
1
85006

1268
1
family

1
genus
32207

172042

SRS052876\_contig\_number\_49
1
species
